# Neurexins 1–3 Each Have a Distinct Pattern of Expression in the Early Developing Human Cerebral Cortex

**DOI:** 10.1093/cercor/bhw394

**Published:** 2016-12-24

**Authors:** Lauren F Harkin, Susan J Lindsay, Yaobo Xu, Ayman Alzu'bi, Alexandra Ferrara, Emily A Gullon, Owen G James, Gavin J Clowry

**Affiliations:** 1 Institute of Neuroscience, Newcastle University, Framlington Place, Newcastle upon Tyne NE2 4HH, UK; 2 Institute of Genetic Medicine, Newcastle University, International Centre for Life, Parkway Drive, Newcastle upon Tyne NE1 3BZ, UK; 3 Present address: School of Healthcare Science, Manchester Metropolitan University, Manchester, M1 5GD, UK; 4 Present address: Wellcome Trust, Sanger Institute, Cambridge, CB10 1SA, UK; 5 Present address: MRC Centre for Regenerative Medicine, University of Edinburgh, Edinburgh, EH16 4UU, UK

**Keywords:** cortical development, neurexins, neurodevelopmental disorders, subplate

## Abstract

Neurexins (NRXNs) are presynaptic terminal proteins and candidate neurodevelopmental disorder susceptibility genes; mutations presumably upset synaptic stabilization and function. However, analysis of human cortical tissue samples by RNAseq and quantitative real-time PCR at 8–12 postconceptional weeks, prior to extensive synapse formation, showed expression of all three *NRXN*s as well as several potential binding partners. However, the levels of expression were not identical; *NRXN1* increased with age and *NRXN2* levels were consistently higher than for *NRXN3*. Immunohistochemistry for each NRXN also revealed different expression patterns at this stage of development. NRXN1 and NRXN3 immunoreactivity was generally strongest in the cortical plate and increased in the ventricular zone with age, but was weak in the synaptogenic presubplate (pSP) and marginal zone. On the other hand, NRXN2 colocalized with synaptophysin in neurites of the pSP, but especially with GAP43 and CASK in growing axons of the intermediate zone. Alternative splicing modifies the role of NRXNs and we found evidence by RNAseq for exon skipping at splice site 4 and concomitant expression of KHDBRS proteins which control this splicing. NRXN2 may play a part in early cortical synaptogenesis, but NRXNs could have diverse roles in development including axon guidance, and intercellular communication between proliferating cells and/or migrating neurons.

## Introduction

Neurodevelopmental conditions such as autism spectrum disorders (ASDs) schizophrenia and intellectual disability are highly heritable and can be regarded as disorders in formation of complex neural circuitry which, although specified by genetic instructions, relies on adaptive mechanisms to fine tune connectivity. A large number of candidate susceptibility genes have been identified (Pinto et al. 2010; Betancur 2011; Cukier et al. 2014; Kavanagh et al. 2015) many coding for proteins that interact in synapse formation, stabilization and function, including neurexins (NRXNs) as well as their postsynaptic binding partners (Ye et al. 2010; Reichelt et al. 2012; Bang and Owczarek 2013) focusing attention on the synapse and aberrant circuitry (Südhof 2008). However, synapses and their proteins are ubiquitous in the brain, whereas circuit alterations identified *postmortem*, or by fMRI and EEG, suggest localized deficits in the frontal and temporal lobes of the cerebral cortex in particular, as well as in the cerebellum and basal ganglia (Courchesne et al. 2007; Miyata et al. 2009; Kubota et al. 2013; Catani et al. 2016). Perhaps selected circuits are particularly sensitive to synaptic dysfunction. Alternatively, uncovering susceptibility gene expression patterns during early development may suggest additional roles key to understanding these localized deficits.

The NRXN genes are among the largest in the genome, exceeding 1 Mb, and differentially spliced into thousands of isoforms (Missler and Südhof 1998; Rowen et al. 2002; Schreiner et al. 2014). Each NRXN gene codes for a longer α and shorter β transcript transcribed from separate promoters (Ushkaryov et al. 1994). The α transcript contains six laminin/NRXN/sex-hormone binding globulin (LNS) domains and three epidermal growth factor (EGF)-like domains while β transcripts have the same transmembrane and cytoplasmic domain but only the sixth LNS domain (Ullrich et al 1995; Missler and Südhof 1998). Six differential splicing sites have been identified in α *NRXN*s (splice sites 1–6) two of which, 4 and 5, are also present in β *NRXN*s (Schreiner et al. 2014; see Supplementary Fig. 1 ).

α NRXNs are involved in Ca2+-dependent neurotransmitter release (Missler et al. 2003) their intracellular PDZ domain binding to proteins such as calcium/calmodulin-dependent serine protein kinase (CASK) which couple NRXN-mediated cell adhesion to synaptic vesicle exocytosis machinery (Hata et al. 1996). However, they are not required for synapse formation (Missler et al. 2003). β NRXNs also recruit other PDZ domain proteins to the presynaptic membrane but chiefly bind to neuroligin (NLGN) proteins across the synaptic cleft via their extracellular domain (Ichtchenko et al. 1995; Koehnke et al. 2010) as do certain α NRXN splice variants (Boucard et al. 2005; Reissner et al. 2013). These events are regulated by alternative splicing of *NRXN* genes (Ichtchenko et al. 1995; Ichtchenko et al. 1996). Different roles in synaptogenesis and synaptic transmission have been attributed to α and β NRXNs, due to separate ligand interactions (Petrenko et al. 1996; Reissner et al. 2013).

Binding affinities differ between various pairs of NRXNs and NLGNs, controlled by alternative splicing of both binding partners (Comoletti et al. 2006). In vertebrates, NRXNs (and NLGNs) are synthesized throughout the brain in all excitatory and inhibitory neurons (Ichtchenko et al. 1995, 1996; Ullrich et al. 1995). The different α- and β NRXNs are coexpressed in the same class of neuron (Ullrich et al. 1995); however, each type of NRXN (1, 2, or 3; Ullrich et al. 1995) and also different α splice variants, mRNA and protein (Schreiner et al. 2014; Schreiner et al. 2015) are differentially distributed between different neuronal types. In rodents, the STAR (Signal Transduction and Activation of RNA) proteins KHDBRS1, 2, and 3 (also known as SAM68, SLM1, and SLM2/T-STAR, respectively) regulate alternative splicing of *Nrxns* (Iijima et al. 2011; Klein et al. 2013; Ehrmann et al. 2013) and determine affinity for different binding partners (Vuong et al. 2016).

Numerous studies have found deletions, truncations, and copy number variants in *NRXN* genes at a higher frequency in neurodevelopmental disorders than in the control groups. The evidence is strongest for *NRXN1*. Two original studies detected both NRXN1β gene variants (Feng et al. 2006) and α gene variants (Friedman et al. 2006) in ASD patients. Subsequently multiple studies have found evidence for *NRXN1* as a candidate susceptibility gene for ASD, schizophrenia and intellectual disability (Szatmari et al. 2007; Kim et al. 2008; Kirov et al. 2008; Glessner et al. 2009; Rujescu et al. 2009; Ching et al. 2010; Gauthier et al. 2011; Viñas Jornet et al. 2014). Mutations in *NRXN2* and *3* have also been implicated although the evidence is less compelling; an identified *NRXN2* gene mutation was found to result in a truncated protein which did not bind its NLGN partner in vitro (Gauthier et al. 2011) and there is supporting evidence from genome wide association studies (Cukier et al. 2014). *NRXN3* mutations have been detected in autistic children from four families (Vaags et al. 2012) and a subsequent association study linked *NRXN3* polymorphisms with schizophrenia (Hu et al. 2013). In mice knock-out of *Nrxn*1α-encoded isoforms targeting the first coding exon resulted in a mild ASD-related phenotype (Etherton et al. 2009; Grayton et al. 2013). Knock-out of *Nrxn2* exon 23, shared by *α* and *β* isoforms, resulted in deficits in social interaction, social memory and anxiety-like behavior (Dachtler et al. 2014; Born et al. 2015).

Preliminary studies suggest that *NRXNs* and associated genes are all expressed at the earliest stages of cortical plate (CP) formation (Ip et al. 2010; Kang et al. 2011) when very few synapses are present in the human cerebral cortex (Kostović and Rakic 1990). This suggests additional roles for NRXNs although an alternative (but not mutually exclusive) explanation would be that perhaps correct functioning of precocious synaptic circuitry present in the presubplate (pSP) and marginal zone (MZ) is crucial to cortical development. To explore these possibilities further, we have studied the expression of the three *NRXN* genes at these early stages and provided additional information on the expression of NRXN binding partners, and regulators of *NRXN* alternative splicing, to gain a more complete picture of how mutations in *NRXNs* may perturb human cortical development. Some of these findings have already been published in abstract form (Harkin et al. 2015).

## Methods and Materials

### Human Tissue

Human fetal brain samples between 8 and 12 postconceptional weeks (PCWs) were obtained from the MRC-Wellcome Trust Human Developmental Biology Resource (HDBR, http://www.hdbr.org; Gerrelli et al 2015). Brains were collected from terminations of pregnancy with maternal written consent and approval from the Newcastle and North Tyneside NHS Health Authority Joint Ethics Committee. Age was determined by the assessment of external morphology (O'Rahilly et al 1987; Bullen and Wilson 1997) and by comparing foot and heel to knee length to a standard growth chart (Hern 1984). Adult brain sections were obtained from the Newcastle Brain Tissue Resource (NBTR). Brains were collected with written consent from donors postmortem with approval from the Newcastle and North Tyneside NHS Health Authority Joint Ethics Committee. Both the HDBR and NBTR are regulated by the UK Human Tissue Authority (HTA; www.hta.gov.uk) and operate in accordance with the relevant HTA Codes of Practice.

**Figure 1.**
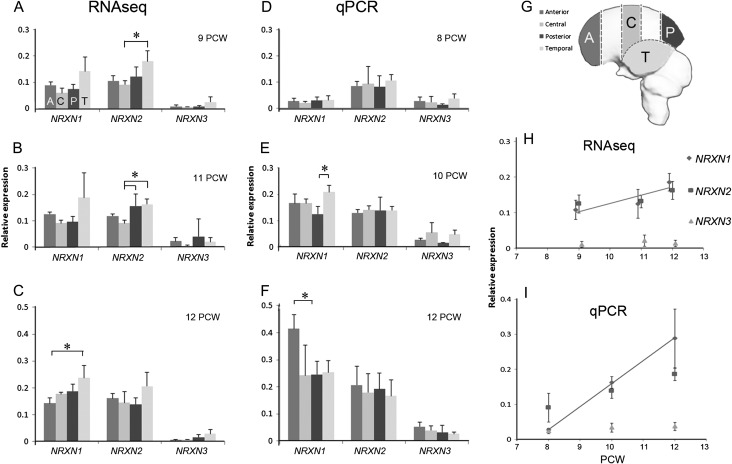
The differences in *NRXN* expression with age and cortical region. (*A*–*C*) Expression by RNAseq relative to reference genes and (*D–F*) expression by qPCR (relative to the same reference genes), in four different cortical regions; anterior (A), central (C), posterior (P), and temporal (T) at different ages postconception. (*G*) The precise locations of the four sampling regions. Error bars represent standard errors of the mean. Statistically significant differences in expression between regions at a given age are marked with an asterisk (one-way ANOVA, *P* < 0.05). RNAseq and qPCR data revealed broadly the same expression patterns with a slight tendency toward increased expression in the temporal cortex at all ages. (*H* and *I*) Changes in expression with age for all pooled cortical samples. Blue lines mark statistically significant positive correlations showing *NRXN1* expression increases with age. *NRXN2* expression remains relatively high throughout, *NRXN3* expression relatively low.

Whole fetal brains were isolated from the skull and the meninges were removed. The hemispheres were split apart allowing removal of the choroid plexus and subcortical structures leaving only the cerebral cortex. One or both hemispheres were then divided into six sections. Each hemisphere represented an independent sample. The temporal lobe, including lateral and medial walls was removed and labeled section 6. The remaining cortex was divided into five sections of equal width from the anterior (A) to the posterior (P) pole of the cortex including lateral and medial cortical walls (labeled 1–5). Sections 1, 3, 5, and 6 were used for RNA extraction and corresponded to anterior, central (C), posterior and temporal (T) regions (see Fig. 1). Sections were immediately frozen and stored at –80°C and subsequently used to extract RNA. Other fetal brains were fixed in buffered 4% paraformaldehyde solution (Sigma Aldrich, Dorset, UK) and embedded in paraffin wax before sectioning.

### RNA Extraction and Reverse Transcription

RNA was extracted from sections 1 (A), 3 (C), 5 (P), and 6 (T) using the QIAgen RNeasy mini kit (Qiagen, Manchester, UK) according to the manufacturer's protocol. Sixty-three samples were taken in all at 9, 11, and 12 PCW (see Table 1). The concentration and quality of RNA was measured using the Nanodrop 8000 (Thermo Fisher Scientific, Cramlington, UK) to detect spectrophotometric absorbance at 260 nm. To control for RNA purity and degradation, 260/230 and 260/280 spectrophotometric ratios were only accepted between the ranges of 1.8 and 2.1. A selection of the RNA was run on the Bioanalyser 2100 (Agilent, Stockport, UK) and all RIN values measured were above 7.

**Table 1:** A summary of the number of tissue samples taken at each age and location for either RNAseq analysis or qPCR

Age (PCW)	Number of samples				
	Anterior	Central	Posterior	Temporal	Total
RNAseq					
9	4	3	4	8	19
11	4	3	4	8	19
12	5	3	6	10	24
					62
qPCR					
8	5	5	5	3	18
10	5	5	5	3	18
11	5	5	5	5	20
					56

At each age/location samples were taken from a minimum of two brains as samples taken from each hemisphere were counted separately.

To reverse transcribe the RNA to cDNA, 2 µg of random primers (Promega, Southhampton, UK), up to 5 µg of RNA, and 1 µl dNTPs (10 mM) were combined and the volume made up to 13 µl using RNase free water. The mixture was heated at 65°C for 5 min and cooled on ice for a minimum of 1 min. Four microliters 5× first strand buffer, 1 µL (200 U/µL) Superscript Reverse Transcriptase (Invitrogen, Thermo Fisher Scientific), 1 µL of 1 M dithiothreitol and 1 µL RNaseOut Recombinant Ribonuclease Inhibitor (Invitrogen, 40 U/µL) were added to the same tube and incubated at 25°C for 5 min before being heated to 50°C for 60 min. The reaction was inactivated by heating to 70°C for 15 min and the cDNA was stored at –20°C. Total cDNA concentration was measured using the Nanodrop 8000 (Thermo Fisher Scientific) to detect spectrophotometric absorbance at 260 nm and 260/230, 260/280 spectrophotometric ratios were between the accepted range of 1.7–2.1. cDNA samples were then diluted to a concentration of 100 ng/µL for use in PCR.

### RNAseq Methodology

Full details of the origins, collection, preparation, sequencing, and analysis of the human fetal RNA samples are provided by Lindsay et al. (2016). The entire RNAseq dataset from which data were extracted for this study has been deposited at www.ebi.ac.uk/arrayexpress/experiments/E-MTAB-4840. Briefly, cDNA was generated from the RNAs using Illumina's Stranded mRNA Sample Prep Kit. Four hundred nanograms of total RNA was used as the input for each sample. The concentration of each library was determined using the KAPA quantitative real-time PCR (qPCR) kit (KK4835) and triplicate reactions using three independent 106-fold dilutions of the libraries. The size profile of approximately 15% of the libraries was evaluated using an Agilent Bioanalyzer DNA 1000 chip. The average final library size was between 272 and 467 bp (includes 120 nucleotides of adapter sequence). The libraries were sequenced on an Illumina HiSeq2000. The high-quality reads were then mapped to the human reference genome hg38 with Tophat2 (Kim et al. 2013). Reads aligned to genes and exons were counted with BEDTools (Quinlan and Hall 2010). Read length was 101 bp prior to trimming and, in the majority of cases, 85 bp after trimming with no reads of <20 bp retained. The minimum number of reads examined per sample was 63 million and the average was 90 million. Differentially expressed genes and exons were then identified with the Bionconductor package DESeq2 (Love et al. 2014).

### Quantitative Real-Time PCR

In order to validate the RNAseq findings, the expression levels of the three *NRXN* genes and three reference genes (*β-ACTIN, GAPDH*, and *SDHA*) (Vandesompele et al. 2002) were measured by qPCR from 56 samples taken from the four sampled sections at 8, 10, or 12 PCW (see Table 2). Primers were designed according to the standard criteria for PCR primer design using the Primer3 (v. 0.4.0) designing program (http://frodo.wi.mit.edu/primer3/). Primers synthesis and product sequencing for quality control were performed by Eurofins MWG Operon1 (http://www.eurofinsgenomics.eu). Table 2 presents the sequence and product size of each primer used. A SYBR Green-based rtPCR assay was performed in 7900HT Fast Real-Time PCR system (Applied Biosystems, Warrington, UK). A total volume of 10 μL qPCR reaction was set up in triplicates, containing 5 μL of 2× SYBR Green qPCR Master Mix (Invitrogen), 1 μL of the diluted cDNA template, 0.5 μL of each primer (10 ρmol/μL), and 3 μL of Molecular Biology grade water. A negative control was incorporated by replacing the cDNA template with Molecular Biology grade water (VWR International, Lutterworth, UK). A standard thermal cycle protocol was used as previously described (Ip et al. 2010). The results of each reaction were analyzed by uploading raw data to the Real-Time PCR Miner web site (http://www.miner.ewindup.info). This calculated the reaction efficiency and the fractional cycle number at threshold (CT) for each reaction (Zhao and Fernald 2005).

**Table 2:** List of primers for qPCR

Gene	Forward primer 5′–3′	Reverse primer 5′–3′	Amplicon size (bp)
NRXN1	aggacattgacccctgtgag	ccttcatcccggtttctgta	205
NRXN2	catcctcctctacgccatgt	ttgttcttcttggccttgct	165
NRXN3	gctgagaacaaccccaata	atgctggctgtagagcgatt	179
*BACTIN	ctacaatgagctgcgtgtggc	caggtccagacgcaggatggc	271
*GAPDH	tgcaccaccaactgcttagc	ggcatggactgtggtcatgag	87
*SDHA	tgggaacaagagggcatctg	ccaccactgcatcaaattcatg	86

*Reference genes BACTIN, β-actin; GAPDH, glyceraldehyde-3-phosphate dehydrogenase; SDHA, succinate dehydrogenase complex subunit A.

### Quantitative Comparison of RNAseq and qPCR Data, and Individual Exon Expression by RNAseq

To make expression data comparable between different methodologies (Al-Jaberi et al. 2015), we divided the expression level of the gene of interest, Reads Per Kilobase of transcript per Million mapped reads (RPKM) for RNAseq, and CT values for qPCR, by the geometric mean expression of three reference genes *(β-ACTIN, GAPDH*, and *SDHA*; Vandesompele et al. 2002) to give relative expression levels in different regions of the cortex at different ages which were then compared by one-way ANOVA followed by Tukey's post hoc test. Expression of the three reference genes was found, on average, to change little across the different regions of the cortex or with age, by either RNAseq or qPCR (Supplementary Fig. 2) validating the choice of these genes.

Expression levels of NRXN-related genes, KHDBRS genes, and individual NRXN exons (6, 17, 19, and 20; see Supplementary Fig. 1) were quantified in terms of normalized RPKM per exon for between 16 and 33 samples for each gene from across the cortex, right, and/or left hemisphere from 3 fetuses at 9 PCW, 5 fetuses at 11 PCW, and 4 fetuses at 12 PCW. The differences in expression between genes or exons were tested by one-way ANOVA followed by Tukey's post hoc test.

### Immunohistochemistry

Deparaffinised coronal and sagittal sections collected on slides were immersed in 1.5 % hydrogen peroxide/methanol solution (Merck Millipore, Watford, UK) to block activity of endogenous peroxidases followed by heat-mediated antigen retrieval treatment in citrate buffer (pH 6.0) before incubating sections with 10% of the appropriate normal serum (Vector Laboratories, Peterborough, UK) in Tris-buffered saline (TBS) for 10 min. For immunoperoxidase, staining sections were then incubated with a primary antibody (see Table 3 for sources of antibodies) in TBS solution overnight at 4°C. Sections were then washed and incubated for 30 min with the appropriate biotinylated secondary antibody (Vector Laboratories, Peterborough, UK), washed then incubated for 30 min with Vectastain Elite ABC kit (Vector Labs) and developed using 3,3′-diaminobenzidine (Vector Labs). Sections were dehydrated, cleared, and coverslipped.

**Table 3:** Primary antibodies employed

Antigen	Supplier	Previous use in human	Dilution	Species
NRXN1α+β	Santa Cruz. Sc14334	Western blot: Kim et al. (2008).	1/300	Goat polyclonal
		Immunohistochemistry: Zhang et al. (2013).		
NRXN2α	AbCam. Ab34245	Immunohistochemistry: Borsics et al. (2010).	1/2000	Rabbit polyclonal
NRXN3α+β	Sigma-Alldrich Prestige. HPA 002727	Tested by human protein atlas including immunohistochemistry.	1/300	Rabbit polyclonal
CASK	AbCam. ab126609	Immunohistochemistry: Wei et al. (2014).	1/350	Rabbit monoclonal
KHDBRS1	Santa Cruz. Sc333		1/1500	Rabbit polyclonal
KHDBRS2	Dr Peter Scheiffele		1/10 000	Rabbit polyclonal
KHDBRS3	Dr Peter Scheiffele		1/5000	Guinea pig polyclonal
PAX6	Covance, PRB-278P	Immunohistochemistry: Bayatti et al. (2008).	1/1500	Rabbit polyclonal
TBR1	AbCam Ab31940	Immunohistochemistry: Bayatti et al. (2008).	1/1500	Rabbit polyclonal
GAP43	Sigma-Alldrich G9264	Immunohistochemistry: Bayatti et al. (2008).	1/10 000	Mouse monoclonal
SYP	Sigma-Alldrich	Immunohistochemistry: Bayatti et al. (2008).	1/1000	Mouse monoclonal

For antigenic sites of anti-NRXN antibodies, see Supplementary Fig. 1. anti-NRXN1 has also been tested against recombinant mouse NRXN1 expressed in a human kidney cell line (Cheng et al. 2009).

For immunofluorescent double labeling, including using two antibodies from the same species, the following method was employed (Goto et al. 2015; Harkin et al. 2016). Sections were incubated with the first primary antibody as before, washed and incubated with the appropriate ImmPRESS HRP IgG (Peroxidase) Polymer Detection Kit (Vector Labs) for 1 h, washed then incubated for 30 min with tyramide signal amplification (TSA) fluorescein plus system reagent (Perkin Elmer, Buckingham, UK). After washing but before application of a second primary antibody, sections were subjected to heat-mediated antigen retrieval in boiling citrate buffer (pH 6.0). This removed the first set of primary and secondary antibodies employed but left fluorescent tyramide covalently bound to the tissue section. The method above was repeated for the detection of this second primary antibody except that TSA rhodamine plus was used for detection. Sections were then washed, counterstained with 4′,6-diamidino-2-phenylindole, dihydrochloride (Thermo Fisher Scientific) and coverslipped with Vectashield (Vector Labs).

## Results

### Quantitative Analysis of mRNA Reveals Distinct Patterns of *NRXN* Gene Expression

Sixty-three samples of RNA, taken from four different regions of the cortical surface (see Fig. 1*G*) at either 9, 11, or 12 PCW were sequenced and mapped. All three *NRXNs* were expressed in all regions and at all ages, although *NRXN3* was consistently expressed at lower levels than *NRXN 1* and *2* (Fig. 1*A*–*C*). *NRXN 1* and *2* were found to be highly expressed (top quartile of all protein coding genes); however, *NRXN3* was expressed at levels we would still expect to be detectable by histological methods (Al-Jaberi et al. 2015). For all three *NRXNS,* at all ages, expression tended to be higher in the temporal cortex, but this only reached statistical significance for *NRXN2* at 9 PCW and 11 PCW compared to central regions of the cortex (Fig. 1*A*,*B*) and for posterior cortex compared to central cortex at 11 PCW (Fig. 1*B*). For *NRXN1,* at 12 PCW, expression was also significantly higher in temporal cortex compared to anterior cortex (Fig. 1*C*). In order to confirm these results, a separate set of 56 samples of RNA were taken for qPCR analysis, although at slightly different age points (8, 10, and 12 PCW, Fig. 1*D*–*F*).

Results from qPCR very largely confirmed the findings by RNAseq except that increased expression in the temporal cortex was not as marked, although for NRXN1 it was significantly higher than expression in the posterior cortex at 10 PCW (Fig. 1*E*). Somewhat anomalously, expression of *NRXN1* in the anterior cortex was markedly high at 12 PCW (Fig. 1*F*). What was apparent from the qPCR study, with its wider age range, was that whereas expression of *NRXN2* remained consistently high over time, *NRXN1* expression increased with age. Results from all regional samples were pooled and plotted against age for both RNAseq and qPCR. It was clear that in both cases, *NRXN* one expression increased significantly with age (Fig. 1*H*,*I*). However, *NRXN2* expression remained constantly high, and *NRXN3* expression consistently lower.

In summary, there were no marked differences in expression between different regions of the cortex detected by either RNAseq or qPCR although expression tended to be higher in temporal cortex, and to a lesser extent frontal cortex, than in central or posterior regions. Although it has been suggested that fronto-temporal circuitry may be primarily “at risk” in neurodevelopmental conditions (Scott-Van Zeeland et al. 2010; Catani et al. 2016), we have found no strong evidence that NRXNs are predominantly expressed there, such that mutations in *NRXN* genes might have a more a deleterious effect in these cortical regions.

## Expression of NRXN-Associated Genes

RNAseq data were then analyzed to determine the levels of expression of proteins associated with NRXNs at synapses, many of which are also candidate susceptibility genes for neurodevelopmental disorders (Ye et al. 2010; Bang and Owczarek 2013). We categorized them according to their location at the synapse, the level of expression and developmental stage (see Table 4). We found that many, but not all, were expressed at similarly high levels as *NRXN*s *1* and *2*, suggesting that they could be potential binding partners for *NRXN*s in the developing cortex. Expression of postsynaptic binding partner *NLGN2* was twice that of *NRXN*s *1* and *2* (Table 4 and data not shown). *NGLN3* and *leucine-rich repeat transmembrane 2* (*LRRTM2*) were highly expressed and *NGLN1* increased in expression with age. *LRRTM*s *3* and *4*, *NLGN4Y* and *X* were moderately expressed but *LRRTM1* showed lower expression. At the presynaptic membrane *cerebellin 1 (CBLN1)* was highly expressed at the earlier stage. Genes associated with the postsynaptic density, *Disks large homolog 4* (*DLG4)* and *SH3 and multiple Ankyrin repeat domains 1 (SHANK1*) were highly expressed although *SHANK2* and *SHANK3* exhibited moderate levels of expression. Genes normally associated with synaptic vesicles, synaptophysin (*SYP*) and *synapse-associated protein 25 kDa* (*SNAP25*) were very highly expressed and *CASK*, which connects neurexins to the exocytotic machinery, also showed high levels of expression similar to *NRXNs 1 and 2* (Table 4).

**Table 4:** Summary of expression of mRNA for neurexins and associated proteins

Protein location	Expression (normalized RPKM)	9 PCW	11–12 PCW
Presynaptic/extracellular	High: 5–25% (40–160)	CBLN1	NRXN1
		NRXN1	NRXN2
		NRXN2	
	Moderate: 25–50% (10–40)	NXPH1	NXPH1
			CBLN1
			NRXN3
	Low: 50–75% (0.4–10)	CBLN2	CBLN2
		NRXN3	
Presynaptic/intraterminal	Very high: top 5% (>160)	SNAP25	SNAP25
		SYP	SYP
	High: 5–25% (40–160)	CASK	CASK
Postsynaptic/extracellular	Very high: top 5% (>160)	NLGN2	NLGN2
	High: 5–25% (40–160)	LRRTM2	LRRTM2
		NLGN3	NLGN1
			NLGN3
	Moderate: 25–50% (10–40)	LRRTM3	LRRTM3
		LRRTM4	LRTMM4
		NLGN1	NLGN4X
		NLGN4X	NLGN4Y*
		NLGN4Y*	
	Low: 50–75% (0.4–10)	LRRTM1	LRRTM1
Postsynaptic/intracellular	Very high: top 5% (>160)	DLG4	DLG4
	High: 5–25% (40–160)	SHANK1	SHANK1
	Moderate: 25–50% (10–40)	SHANK2	SHANK2
		SHANK3	SHANK3

Protein location describes its location in the mature central nervous system. Extracellular means at least part of the molecule is an extracellular domain. Expression is the mean across all cortical regions. Percentage values indicate into which quartile range for all protein coding expression data the expression values fall. RPKM values are indicative and not exact. *NLGNY means from male samples only, female samples gave mean expression 0.11 range 0.07–0.45. NXPH, neurexophilin.

### Localization of Expression of NRXN Proteins by Immunohistochemistry

**Figure 2.**
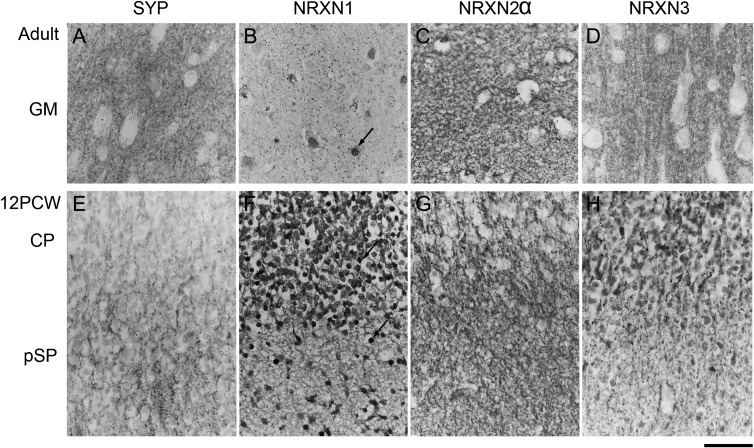
Immunohistochemistry for SYP and the NRXNs in paraffin sections from adult (*A–D*) and fetal (*E–H*) cerebral cortex. (A) Punctate SYP immunoreactivity confined to presumptive synaptic terminals in the gray matter neuropil. In the fetal cortex (E) such punctate staining is largely confined to the pSP. All three NRXNs also exhibited punctate staining (B*–*D) in adult cortex, although at a lower density for NRXN1 with evidence of nuclear staining (arrow, B). In the fetal cortex, NRXN1 was expressed in cell bodies, processes, and some nuclei (arrows) of many immunoreactive cells in the pSP and CP but without punctate staining (F). NRXN2, however, exhibited punctate staining in the pSP in particular (G). NRXN3 was expressed in cell bodies and processes mostly in the CP, with some punctate immunoreactivity, largely in the pSP (H). Scale bar = 50 µm.

The specificity of our antibodies was first tested by immunostaining sections of adult human cerebral cortex in comparison with SYP immunoreactivity. Our antibodies to NRXN1 and three were generated against peptide sequences common to both α and β isoforms, but the antibody to NRXN2 was raised against the α form only (Supplementary Fig. 1). All anti-NRXN antibodies and anti-SYP produced immunoreactivity in gray matter neuropil which was generally punctate in appearance, suggestive of presynaptic terminals and/or synaptic contacts (Fig. 2*A–D*). However, the density of punctate staining was lower for NRXN1 and there was additional immunoreactivity in many cell nuclei. NRXN2α immunoreactivity also appeared in the cytoplasm of some cells including oligodendrocyte-like cells in the white matter (not shown). The density of immunoreactivity was greatly reduced in the white compared to gray matter for all four proteins (not shown). In sections of 12 PCW fetal cortex, SYP immunoreactivity revealed a small amount of neuropil in the pSP likely to contain the earliest synapses (Fig. 2*E*; Bayatti et al. 2008). Only NRXN2α immunoreactivity was strongly expressed in the neuropil of this region (Fig. 2*G*). Both NRXN1 and NRXN3 were expressed in the cell bodies, their immediate processes and, particularly for NRXN1, some nuclei of many immunoreactive cells in the pSP and the overlying CP. Neuropil staining was very weak for NRXN1 but some punctate staining in the pSP and CP was detectable for NRXN3. Because of the unexpected nuclear labeling observed with anti-NRXN1 antibody, we tested immunostaining with the antibody in the presence of blocking peptide according to the manufacturer's instructions (Santa Cruz). All immunostaining was abolished suggesting that the NRXN1 antigenic site was present in the nucleus (Supplementary Fig. 3).

**Figure 3.**
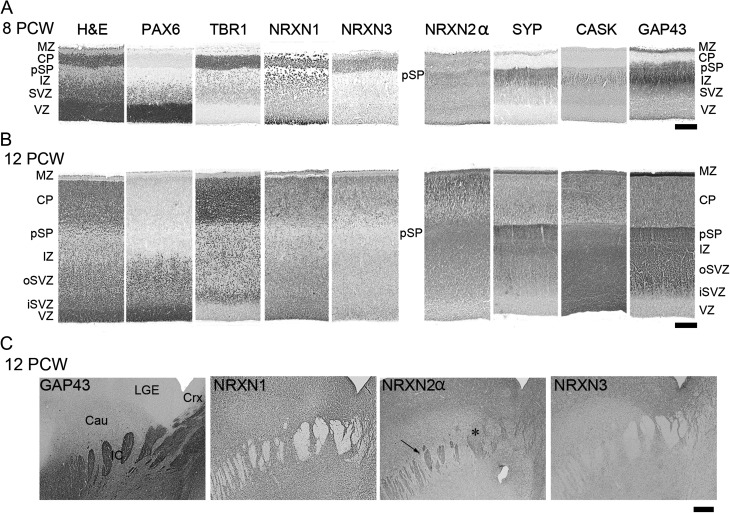
A comparison of NRXN immunoreactivity with other cell markers across the cortical wall at 8 PCW (*A*) and 12 PCW (*B*). H&E, hemotoxylin and eosin stained; o, outer; i, inner. Note that PAX6 revealed radial glial progenitor cells; TBR1, postmitotic neurons; SYP, CASK and GAP43 neurites of the pSP and IZ. NRXN1 and 3 predominantly localized to layers with a high-cellular density, whereas only NRXN2α predominantly colocalized with synaptogenic zones and growing axons. (*C*) Internal capsule in the ventral forebrain in which bundles of growing axons were GAP43 positive, NRXN1 and 3 negative, and partially positive for NRXN2α (arrow) although some axon bundles appeared negative (*). Scale bars: A and B, 200 µm; C, 500 µm.

We proceeded to investigate NRXN immunoreactivity over the age range 8–12 PCW in comparison to the expression of other markers for the different cortical layers (Fig. 3). As has been previously demonstrated (Bayatti et al. 2008), PAX6 identifies radial glial cells in the proliferative ventricular (VZ) and subventricular (SVZ) zones, TBR1 immunoreactivity identifies postmitotic neurons in the SVZ, migrating through the intermediate zone (IZ) and in the pSP CP and outer MZ. GAP43 and SYP immunoreactivity are localized to growing axons, growth cones and newly forming synaptic terminals and delineate the MZ and pSP (SYP more than GAP43) and IZ (GAP43 more than SYP).

**Figure 4.**
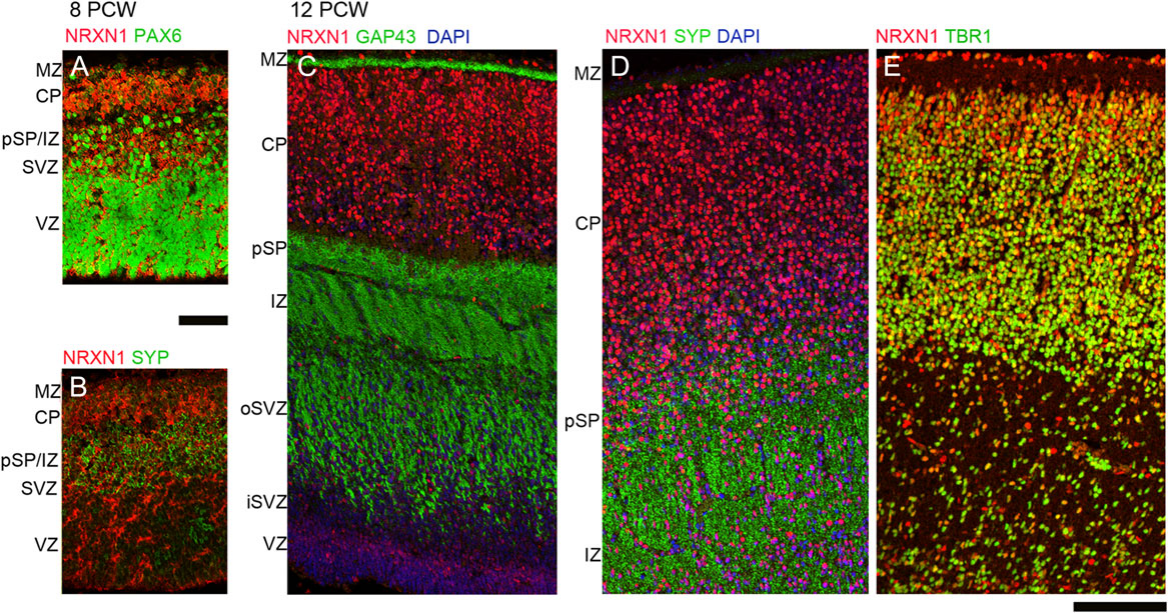
Colocalization of NRXN1 immunoreactivity (red) with phenotypic markers (green) by immunofluorescence. At 8 PCW NRXN1 immunoreactivity was present in cytoplasm/membranes around PAX6-positive radial glia, particularly near the apical ventricular surface (*A*) but showed little colocalization with SYP in the pSP at either 8 PCW (*B*) or 12 PCW (*D*) nor with GAP43 in growing axons of the IZ (*C*). However, there was strong colocalization (orange–yellow) with TBR1 in the postmitotic neurons of the CP. Scale bars = 100 µm.

Each NRXN showed a distinct pattern of immunoreactivity. At 8 PCW, NRXN1 expression was strongest in cells lining the apical surface of the VZ, in the pSP, and in the CP, particularly at the border with the MZ, and in the MZ itself and was predominantly cytoplasmic or membranous, rather than nuclear (Figs 3*A* and 4*A*). Immunofluorescent double labeling revealed NRXN1 expression around the margins of PAX6-positive progenitor cells in the VZ (Fig. 4*A*) but no strong colocalization with SYP in the pSP (Fig. 4*B*). Similarly, by 12 PCW, there was no expression in the network of neurites in the MZ, pSP, and IZ that was strongly immunopositive for GAP43 and SYP (Fig. 4*C*,*D*). However, NRXN1 was expressed in many but not all cell bodies, in all layers of cortical wall and, expression was strongest in the VZ (Figs 3*B* and 4*C*) and in the immature neurons of the MZ and the superficial CP and appeared to be expressed in cell nuclei (Figs 3*B* and 4*D*,*E*). Many cells coexpressed TBR1 and NRXN1 but NRXN1 expression predominated in the superficial CP, TBR1 in the lower CP, with cells expressing one, the other or both in the pSP (Fig. 4*E*). There was no evidence for more intense NRXN1 immunoreactivity in the frontal or temporal lobes (not shown).

**Figure 5.**
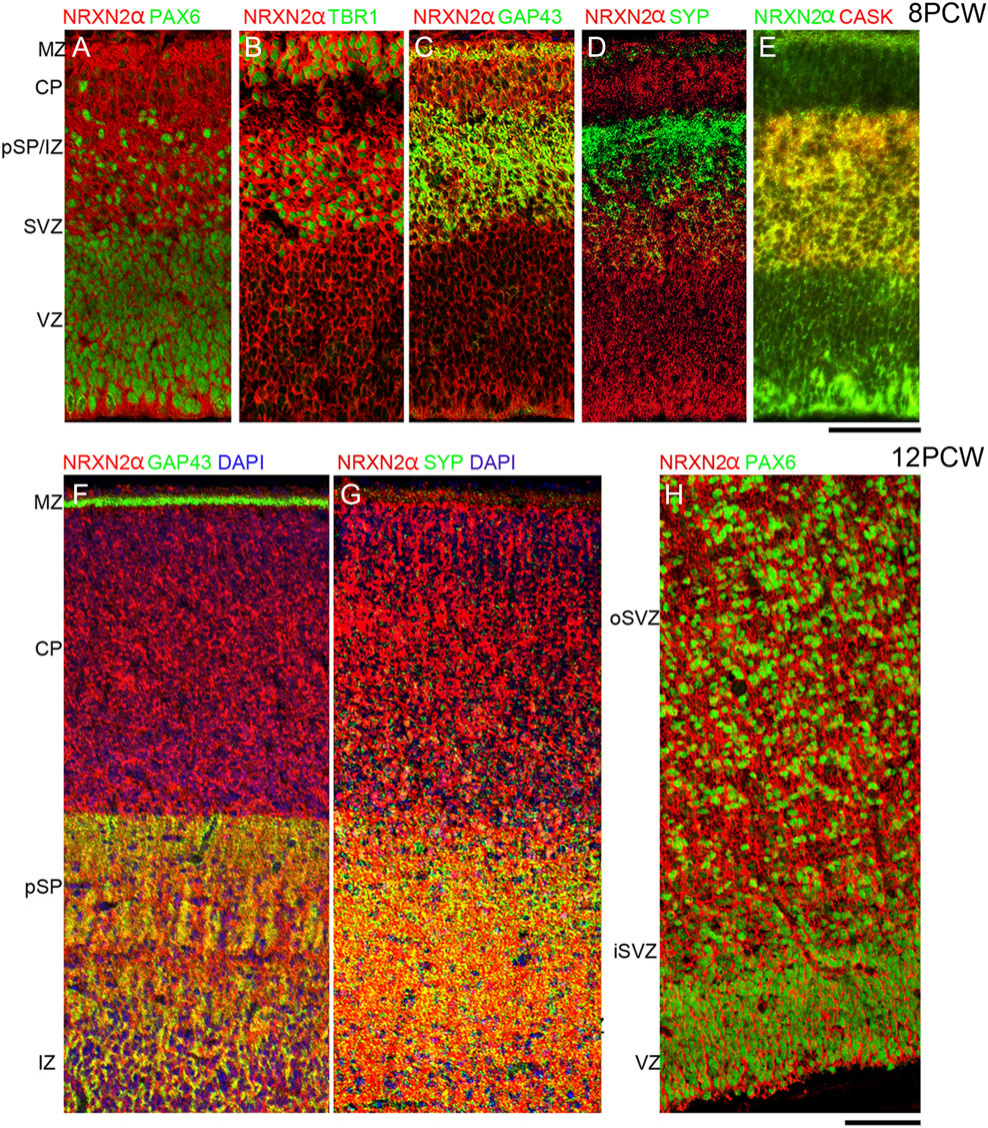
Colocalization of NRXN2 immunoreactivity (red) with phenotypic markers (green) by immunofluorescence. At 8 PCW NRXN2 was ubiquitously expressed and surrounded both PAX6-positive nuclei of progenitor cells (*A*) and TBR1-positive nuclei of postmitotic neurons (*B*) (green). In the pSP and IZ, NRXN2 showed stronger colocalization (yellow) with GAP43 (*C*) and particularly CASK (*E*) than with SYP (*D*). At 12 PCW NRXN2 and GAP43 are strongly colocalized (yellow) in the pSP and IZ but not the MZ (*F*). NRXN2 also colocalized with SYP in pSP and IZ (*G*). As at 8 PCW, many cells coexpressed NRXN2 in the cytoplasm and processes surrounding PAX6-positive nuclei, especially in the oSVZ and VZ (*H*). iSVZ and oSVZ, inner and outer subventricular zone. Scale bars = 100 µm in A–E and F–H.

NRXN2α immunoreactivity was expressed strongly throughout the cortical wall at 8 PCW (Fig. 3A) both in and around cells, but not in their nuclei, and in networks of processes away from the cell bodies. It was colocalized both with PAX6-positive progenitors and TBR1-positive postmitotic neurons (Fig. 5*A*,*B*). Although present in the pSP it showed comparatively weak colocalization with SYP but exhibited stronger expression and colocalization with GAP43, particularly in the IZ (Figs 3*A* and 5*C*,*D*). It was also strongly coexpressed with CASK, particularly in the IZ (Fig. 5*E*). This pattern of expression was little changed by 12 PCW (Fig. 5*F*–*H*) although colocalization with SYP was possibly more evident by this stage. Expression of CASK became much stronger in the proliferative SVZ and VZ at 12 PCW where it was colocalized with all three NRXNs (Fig. 3*B*).

**Figure 6.**
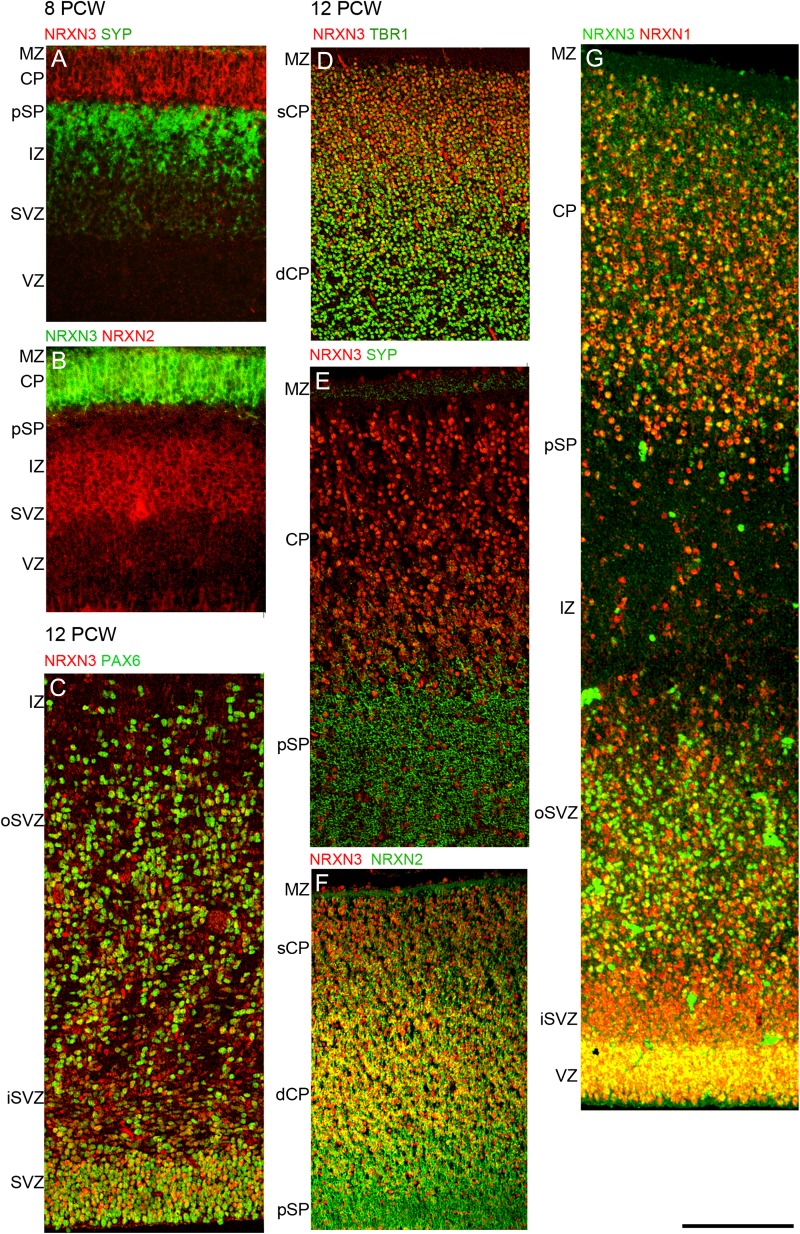
Colocalization of NRXN3 immunoreactivity (red) with phenotypic markers (green) by immunofluorescence. At 8 PCW NRXN3 expression was almost completely confined to the CP and separated from SYP immunoreactivity (*A*) showing only weak colocalization with NRXN2 in the CP (pale green, *B*). At 12 PCW NRXN3 was expressed in the proliferative zones and colocalized (yellow) with PAX6 (*C*) and in the CP was colocalized with TBR1, especially in the deeper layer (dCP) (*D*). NRXN3 did not colocalize with SYP (*E*) but was coexpressed with NRXN2 in the dCP (*F*). NRXN1 and 3 were colocalized throughout the cortical wall at 12 PCW but especially strongly in the CP and VZ (*G*). Scale bar = 200 µm.

At 8 PCW, NRXN3 immunoreactivity was almost entirely confined to the CP and MZ (Figs 3*A* and 6*A*,*B*). By 12 PCW, there was an increase in nuclear staining as was observed for NRXN1. NRXN3 immunoreactivity was also detected in the proliferative zones where it colocalized with PAX6-positive progenitors in the upper part of the VZ in particular (Figs 3*B* and 6*C*) and with NRXN2α in the VZ, and to a lesser extent, in the SVZ (Fig. 6*F*). NRXN2α expression was present at the apical surface of the VZ where NRXN3 was absent. In the CP, NRXN3 expression was colocalized with TBR1, NRXN1, and NRXN 2α (Fig. 6*D*,*F*,*G*) but in all cases NRXN3 expression was relatively stronger in the superficial part of the CP near the border with the MZ, where TBR1 in particular was more weakly expressed (Fig. 6*D*). There was very little colocalization between NRXN3 and SYP in the pSP and MZ, although NRXN3 positive cells were present in these layers (Figs 3*B* and 6*E*).

### Expression of KHDBRS mRNA and Proteins and Alternative Splicing of NRXNs

**Figure 7.**
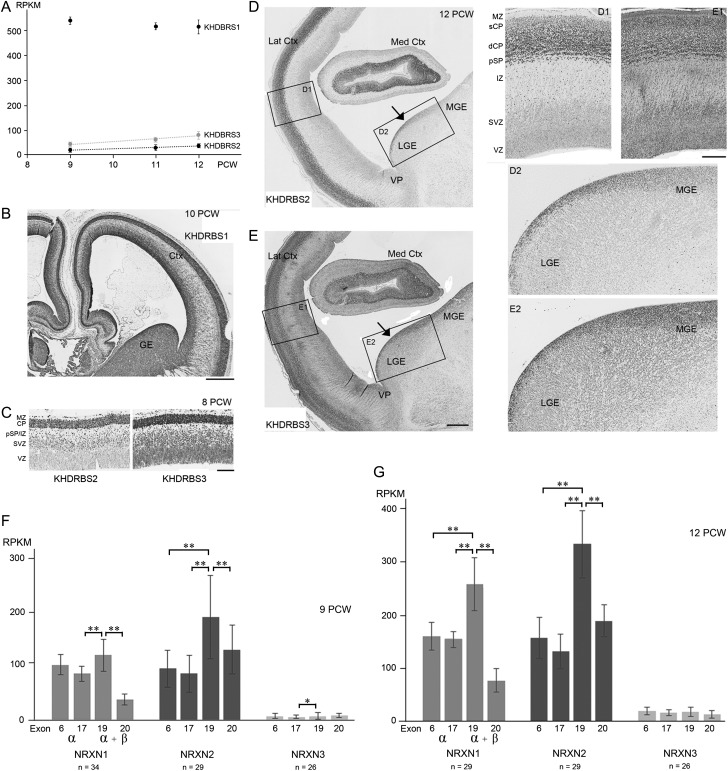
*KHDRBS 1–3* expression in human early fetal forebrain and suppression of NRXN exon 20. (*A*) RNAseq analysis found very high expression of *KHDBRS1* maintained with age, and moderate expression of *KHDRBS2* and *3* which increased with age (dotted trend lines indicate a significant correlation, *P* < 0.001). (*B*) Patterns of KHDRBS immunoreactivity reflected mRNA expression; KHDBRS1 immunoreactivity was intense throughout the telencephalon at 10 PCW. (*C*) At 8 PCW, many KHDBRS2 positive cells were observed in the MZ, CP, and pSP, isolated cells were also seen in the IZ and SVZ; there was stronger immunostaining for KHDBRS3 in the CP, MZ, and pSP/IZ, with widespread moderate immunoreactivity in the SVZ and VZ. At 12 PCW, KHDBRS2 immunoreactive cells were predominant in the deeper part of the CP (dCP), pSP and MZ, but rare in the superficial CP (sCP). Scattered positive cells were observed in the IZ and SVZ along with a small number in the VZ (*D* and *D1*). For KHDBRS3, a complex pattern of immunostaining was observed in the cortex with more immunopositive cells in the sCP and close to the boundary with the pSP, but with a band of weaker immunoreactivity in the middle of the CP. Weak to moderately immunoreactive cells were present throughout the IZ and proliferative layers (*E* and *E1*). KHDBRS2 expression was greater in the proliferative zone of the LGE than the MGE with a few scattered positive cells present in the postmitotic mantle zone (*D* and *D2*). KHDBRS3 immunopositive cells were present throughout the proliferative zones of the LGE and MGE, with scattered cells observed in the postmitotic mantle zone (*E* and *E2*). Med and Lat Crtx, medial and lateral cortex; VP, ventral pallium, border between cortex and ganglionic eminences. (*F* and *G*) Expression of four exons, present in all three *NRXNs,* were chosen for analysis; 6 and 17 that are in the alpha (α) isoforms only of NRXN proteins, and 19 and 20 translated in both alpha and beta (β) isoforms. KHDBRS proteins splice *NRXN* mRNA to exclude exon 20. Statistically significant reductions in exons 6 or 17 compared to 19 indicate less expression of α compared to the β isoforms, and significant reductions in 20 compared to 19 indicates suppression of exon 20 expression by KHDBRS proteins (*P < 0.05, **P < 0.01 by Tukey's post hoc test). Error bars indicate standard errors of the mean. Scale bars = 750 µm for B; 200 µm for C; 1 mm for D and E; 250 µm for D1, D2, E1, and E2.

From the RNAseq data, it can be seen that all three *KHDBRS* genes were expressed in the developing cortex (Fig. 7*A*). *KHDBRS1* showed very high levels of expression, four to five times greater than the highest expression levels measured for *NRXN1* and *2*. *KHDBRS2* and *3* exhibited expression levels comparable to *NRXNs*, although *KHDBRS3* showed slightly higher expression than *KHDBRS2*. There was no significant change in expression levels with age or with cortical location (Fig. 7*A*).

In general terms, protein expression reflected mRNA expression. Immunoreactivity for KHDBRS1 (SAM68) was intense and appeared to be present in every cell, proliferative and postmitotic, and throughout all compartments of the cerebral cortex and ventral telencephalon, between 8 and 12 PCW (Fig. 7*B*). KHDBRS2 (SLM1) showed the lowest levels of immunoreactivity, in concordance with the findings from RNAseq. At 8 PCW most cells of the MZ, CP, and pSP were immunopositive for KHDBRS2. Isolated immunostained cells were also seen in the IZ and SVZ but not the VZ. By 12 PCW, KHDBRS2 immunoreactive cells were predominant in the deeper part of the CP, pSP, and MZ, but rare in the superficial CP. Scattered positive cells were observed in the IZ and SVZ along with a small number in the VZ. In the ventral telencephalon, KHDBRS2 expression was greater in the proliferative zone of the lateral ganglionic eminence (LGE) than the medial ganglionic eminence (MGE) a few scattered positive cells were also seen in the postmitotic mantle zone (Fig. 7*D*).

KHDBRS3 (SLM2/T-STAR) showed higher levels of immunoreactivity than KHDRBS2. At 8 PCW, in addition to strong immunostaining in the CP, MZ, and pSP/IZ, there was widespread moderate immunoreactivity in the proliferative layers (Fig. 7*C*). By 12 PCW, a complex pattern of immunostaining had emerged in the CP, with more immunopositive cells in the superficial layer and also close to the boundary with the pSP, but with a band of weaker immunoreactivity in the middle of the CP. Weak to moderately immunoreactive cells were present throughout the IZ and proliferative layers (Fig. 7*E*). In the ganglionic eminences, immunopositive cells were present throughout the proliferative zones of the LGE and MGE, with scattered cells present in the postmitotic mantle zone (Fig. 7*E*). In the mouse, KHDBRS2 and 3 are expressed in distinct populations of cortical interneurons with KHDBRS2 restricted to a small subpopulation of calbindin positive interneurons (Iijima et al. 2014). The reduced expression of KHDBRS2 compared to KHDBRS3, particularly in the MGE suggests that in human also that KHDBRS proteins may segregate to different interneuron precursors, but this was not explored further. However, the extensive expression of KHDBRS2 protein in the CP differs from the lack of expression of this protein by the glutamatergic neurons of the adult mouse neocortex (Iijima et al. 2014).

Having established expression of KHDBRS proteins we reexamined the RNAseq data at the level of expression of individual exons of the *NRXN* genes in cortical samples at 9 and 12 PCW. Exons 6 and 17, which belong to α *NRXNs* only, and exons 19 and 20, which are found in both α and β isoforms, were chosen. Alternative splicing by KHDBRS proteins occurs at alternative splice site (AS) 4 and results in suppression of expression of exon 20 compared to exon 19 (Rowen et al. 2002). One-way ANOVA established that there were statistically significant differences in expression between exons for all three *NRXN*s (at 9 PCW for *NRXN1*, *P* < 0.0001; *NRXN2*, *P* < 0.001;* NRXN3*, *P* < 0.03; at 12 PCW for *NRXN1*, *P* < 0.0001; *NRXN2*, *P* < 0.0001; *NRXN3*, *P* < 0.04). Post hoc comparisons revealed that at 9 PCW expression of *NRXN1* exon 19 was significantly higher than expression of exon 17 or exon 20 (*P* < 0.01) suggesting that a modest amount of *NRXN1β* was transcribed and there was a significant two-thirds reduction in expression of exon 20 (Fig. 7*F*)**.** By 12 PCW, the differences in expression between exon 19 and exons 6 and 17 were significant (*P* < 0.01) and larger than at 9 PCW, showing that *NRXN1β* expression had increased. Exon 20 continued to show a high degree of suppression of expression (Fig. 7*F*,*G*)**.**

For *NRXN2*, at both ages studied, expression of exon 19 was significantly higher than all the other exons studied (*P* < 0.01). Relative production of *NRXN2β* compared to α isoforms seems to be greater than for *NRXN1 isoforms* especially at 9 PCW, but the degree of suppression of exon 20 is not as marked as for *NRXN1* (Fig. 7*F*,*G*). For *NRXN3*, the expression levels of each exon were low and not markedly different, except that we observed significantly greater expression of exon 19 compared to 17 at 9 PCW; evidence for some production of the *NRXN3β* isoform, at least at this stage of development (Fig. 7*F*,*G*).

## Discussion

This study has confirmed that all three *NRXN* genes show significant levels of expression in the early developing human cerebral cortex prior to extensive synapse formation, as suggested by preliminary gene expression studies (Ip et al. 2010; Kang et al. 2011), and as was previously shown in the mouse by in situ hybridization (Puschel and Betz 1995). Each NRXN had a unique expression pattern, which suggests that they each may have different functions. NRXN1 and 3 protein showed broadly similar patterns of expression, strongest in the CP, although only *NRXN1* expression increased with age whereas *NRXN3* expression was relatively low. This is in partial disagreement with Konopka et al. (2012) who found that *NRXN1* was predominantly expressed in postmitotic cortical cells whereas *NRXN3* was predominantly expressed in proliferating cells in both mid-gestation tissue sections and normal human neural progenitor cells differentiating in vitro. More recently, Jenkins et al. (2015) have demonstrated by qPCR that *NRXN1* expression, in both α and β isoforms, increases in expression from 14 PCW until birth in the prefrontal cortex.

*NRXN2* was more highly expressed at earlier stages than *NRXN1* or *NRXN3* throughout, and although present throughout the cortical wall NRXN2α was the only NRXN to strongly colocalized with neurites in the IZ and synaptogenic pSP. This diversity in expression patterns is unexpected as it is generally believed that there is no functional differentiation between α NRXNs based on “rescue” experiments in transgenic mice (Zhang et al. 2005) although this only probed their function in neurotransmitter release at the synapse. However, there are other respects in which NRXNs 1 and three diverge from NRXN2; a phylogenetic tree for the NRXN protein family demonstrates that NRXNs 1 and 3 are more closely related to each other than either is to NRXN2 (Reissner et al. 2013). Whereas *NRXNs 1 and 3* are unusually long genes, greater than one Mega base pairs (Mbp) in length, the *NRXN2* gene, despite having a very similar number of exons and amino acids in the protein, is only 0.117 Mbp in length (Rowen et al. 2002; Tabuchi and Südhof 2002). The smaller length of NRXN2 may permit transcription to be completed within a cell cycle in dividing cells (Rowen et al. 2002: Reissner et al. 2013) explaining its increased expression compared to NRXNs 1 and 3 at earlier stages of development and in proliferative layers. The largest gene NRXN3, on the other hand, showed the greatest degree of restriction to postmitotic layers.

### Regulation of Alternative Splicing at AS4 and Synapse Formation

It is well established that presynaptic NRXNs on growing axons can interact with NLGNs expressed on growing dendrites to stabilize the postsynaptic site and establish vesicle accumulation at the presynaptic site (Graf et al. 2004; Chen et al. 2010). Although synapses are rare in the early developing cortex, they can be detected by electron microscopy in the MZ and pSP (but not the CP) and increase substantially over the period of the present study (Kostović and Rakic 1990). Proteins associated with synapses such as SYP, vesicular GABA transporter, and CASK have also been detected in the pSP at these ages by immunohistochemistry (Bayatti et al. 2008; Figs 3–6, present study). Therefore, it is reasonable to suppose that NRXN expression could reflect the nascent synaptogenesis occurring at this stage.

Regulation of *Nrxn* exon 20 expression at AS4 generates + or – isoforms that show differential binding to cell-type-specific postsynaptic partners (Baudouin and Scheiffele 2010; Vuong et al. 2016). KHDBRS proteins bring about splicing at AS4 and there is evidence of Khdbrs3 regulated exon skipping during mouse forebrain development (Ehrmann et al. 2013). NRXNβ AS4– isoforms preferentially bind NLGN1 concentrated at glutamatergic synapses, whereas NRXNα AS4– and NRXNβ AS4+ isoforms preferentially bind NLGN2 at GABAergic and glycinergic synapses (Chih et al. 2006). Thus, exclusion of exon 20 in different α or β isoforms might be required to induce formation of both major classes of synapse in the cortex. KHDBRS1 was found to be abundantly expressed in the developing cortex, but in mouse, KHDBRS1 only affects *Nrxn* splicing in the presence of depolarizing neuronal activity (Iijima et al. 2011). As in the 16–20 PCW human cortex, there is only significant neuronal activity in the subplate and the CP is largely quiescent (Moore et al. 2009), it is not expected that KHDBRS1 makes a significant contribution to *NRXN* splicing in our study.

However, both KHDBRS2 and 3 constitutively affect *Nrxn* splicing: ablation of either leads to a severe reduction in the *Nrxn* AS4– isoform in neurons or brain areas where they are typically expressed ( Ehrmann et al. 2013; Iijima et al. 2014; Traunmuller et al. 2014). We observed expression of both these proteins, particularly in the CP and pSP, along with significant reduction in exon 20 expression, which we predict could occur in *NRXN1α* and *NRXN2α* at all ages studied, *NRXN3α* at 12 PCW and also in β isoforms of *NRXN1* and *2* (Fig. 7*F*,*G*). GABAergic synapses are likely to be present in the human pSP at this stage of development (Bayatti et al. 2008) and our data suggest that both NRXNα AS4– and NRXNβ AS4+ isoforms and NLGN2, which interact in their formation (Chih et al. 2006: Vuong et al. 2016) could be expressed in the pSP at this stage of development. NRXN2α immunoreactivity was observed within synaptogenic zones. NRXNα appears important for GABAergic synapse assembly as triple *Nrxnα* knock-out mice show a 50% reduction in cortical GABAergic synapse density (Missler et al. 2003). Early cortical network activity also relies upon glutamate receptor mediated activity (Kilb et al. 2011) and our mRNA expression data suggest the proteins required for glutamatergic synapse formation, NLGN1, NRXN1β AS4–, and NRXN2β AS4– (Chih et al. 2006) could also be present, although their protein expression was not examined in this study.

### NRXNs, Vesicles and Axon Growth

Alpha NRXNs interact with components of the exocytosis machinery indirectly via binding to CASK and synaptotagmin (O'Connor et al. 1993; Hata et al. 1996; Reissner et al. 2013). They are one of the receptors for α-latrotoxin which causes uncontrolled exocytosis (Südhof 2001) and knock-out of *Nrxnα* greatly reduces spontaneous and evoked neurotransmitter release at all synapses in the mouse brain (Missler et al. 2003). Although NRXN2α colocalized with SYP, our marker for presynaptic terminals, in the pSP and MZ, there appeared to be stronger colocalization with GAP43 in the IZ of the cortex dorsally and the internal capsule ventrally (Fig. 3) where growing axons mostly emanating from CP neurons are located at this stage (Ip et al. 2011). There was also strong colocalization of NRXN2α and CASK in growing axons. The fusion of intracellular vesicles with the external membrane at the growth cone provides a mechanism for addition of membrane and guidance receptors to the leading filopodia (Tsaneva-Atanasova et al. 2009) and there is increasing evidence for the role of synapse-related SNARE (soluble *N*-ethylmaleimide-sensitive fusion attachment protein receptor) proteins in the growth of neurites (Kunwar et al. 2010; Zylbersztejn and Galli 2011). NRXN1β has also been shown to promote neuritic outgrowth in concert with NGLN1 and activation of fibroblast growth factor receptor-1 (Gjørlund et al. 2012).

### NRXNs and Other Forms of Intercellular Connections

NRXNs appear not to be just confined to synapses of the nervous system. NRXNs can form a stoichiometric complex with dystroglycan, a ubiquitously expressed transmembrane protein linking cytoskeletal actin to the extracellular matrix. Dystroglycan extracted from brain, heart, and skeletal muscle can interact with NRXN1 (Sugita et al. 2001) and it was found that cardiac isoforms of NRXN3 participate in a complex involving dystroglycan and proteins of the extracellular matrix, presumably involved in intercellular connections (Occhi et al. 2002). Alternative splicing at the α isoform-specific AS2 regulates binding of NRXNαs to dystroglycan (Sugita et al 2001). Dystroglycan expression in the developing mouse cerebral cortex at embryonic day 15, equivalent to the stage of development studied here, is confined to both the CP and the apical surface of the VZ (Lathia et al. 2007) reminiscent of the pattern of NRXN1 immunoreactivity we have observed (Figs 3 and 4). The competitive interaction of dystroglycan and neurexophilin1 with NRXN1α controls the rate of proliferation of human hematopoietic stem cells (Kinzfogel et al. 2011). As radial glial cells predominantly undergo cell division at the apical surface of the VZ (Taverna and Huttner 2010; Harkin et al. 2016) and neurexophilin1 is expressed in the cortex at this stage of development (Table 2), we hypothesize that NRXN1α in particular may interact with the extracellular matrix at tight junctions at the apical surface of the VZ, participating in the control of progenitor cell proliferation, and may also interact with dystroglycan in the CP.

### NRXNs and Cell Migration

Expression of NRXN protein in cells of the VZ, SVZ, and IZ may be an indication of its requirement in cell migration away from the proliferative zones. Indeed, NRXN expression also tended to be higher in the outer part of the CP, where new neurons are arriving, compared to the inner CP where older cells have settled having finished migration (Bystron et al. 2008). Cell adhesion molecules are important for neuronal migration (Maness and Schachner 2007) and molecules such as cadherins, for instance, also found at the synapse in the mature nervous system, are regarded as integral to the migration process (Redies et al. 2012). Certainly, the knock down of *Cntnap2*, which is similar in structure to NRXNs and classed as an autism susceptibility gene, causes abnormal neuronal migration, affecting both cortical projection neurons and interneurons, in transgenic mice (Peñagarikano et al. 2011) resulting in epilepsy and autism-like behavioral deficits. In cases of human *CNTNAP2* gene mutation, cell migration defects, epilepsy, ASD, and language deficits have all been reported (Strauss et al. 2006; Alarcón et al. 2008; Scott-Van Zeeland et al. 2010). The large extracellular portion of CNTNAP2, like the NRXNs (Supplementary Fig. 1), is composed of protein–protein interaction domains including laminin G and EGF repeats ( Poliak et al. 1999). CNTNAP2 interacts extracellularly with CNTN2 (TAG-1) which is expressed early in mouse development and blocking its function results in migration abnormalities of cortical pioneer neurons and GABAergic interneurons (Denaxa et al. 2005; Morante-Oria et al. 2003). Potentially, NRXNs may have as yet unappreciated roles in cell migration, and by extension, neurite outgrowth, processes which largely share the same molecular machinery (Maness and Schachner 2007).

### Nuclear Localization of NRXN 1 and 3

The observation of NRXN-like immunoreactivity in the nucleus was unexpected. This is unlikely to be artefactual as, for NRXN1 at least, it was abolished with blocking peptide. Also the detection of nuclear NRXN expression increased with age which would not be expected for nonspecific staining. Finally, both antibodies employed recognize antigenic sties in the highly conserved c-terminal regions of NRXN1 and NRXN3, rather than sites in the ubiquitous protein domains of the extracellular parts of these molecules. Synaptic proteins are often colocalized to the synapse and the nucleus (Jordan and Kreutz 2009). For instance, CASK is known to translocate to the nucleus where CASK interacts with TBR1 and regulates the TBR1-dependent transcription (Hseuh et al. 2000; Wang et al. 2004). CASK immunoreactivity increased in the CP and proliferative regions at 12 PCW, the same age at which anti-NRXN immunoreactivity increased in these zones. CASK binds indirectly to the c-terminal regions of the NRXNs (Hata et al. 1996). Whether the C-terminal portion of NRXNs translocate to the nucleus, with or without CASK, remains a possibility to be further investigated.

### NRXN Mutations and Neurodevelopmental Disorders

To what extent has uncovering expression patterns during early development suggested roles for NRXNs key to understanding how mutations may cause deficits in brain development? Genetic studies of neurodevelopmental conditions have shown that the great majority of mutations affect NRXN1α expression, leaving β expression intact (Reichelt et al. 2012). We have found that NRXN1 was expressed in substantial amounts in the developing cortex, with a higher proportion likely to be expressed in the α form, especially at 9 PCW where there is protein expression in the VZ. Therefore, mutations in *NRXN1α* in early cortical development could cause subtle alterations in rates and quantity of neuroblast production from radial glia.

A *NRXN2* gene mutation in an ASD patient caused the production of a truncated protein which, in vitro, did not bind its NLGN partner or induce synaptic differentiation (Gauthier et al. 2011). The present study has shown that NRXN2α is the isoform most likely to be involved in induction of synapse differentiation in the human pSP and MZ; therefore, it is plausible that NRXN2 mutations may cause alterations in subplate development. Even at the early stages studied here, synaptic connectivity in the pSP may be generating synchronous oscillatory network activity in a first step toward the subplate developing into a hub for network activity to guide cortical development (Kanold and Luhmann 2010). Disruptions to the subplate have been implicated in epilepsy, ASD, and schizophrenia (Bunney et al. 1997; Kostović et al. 2015; Hutsler and Casanova 2016). However, we have provided equally strong evidence for involvement of NRXN2α in axon outgrowth and pathfinding (see above). It has also been argued recently that dysregulation of axonal growth and guidance should be given greater prominence when considering the etiology of ASD, as many candidate ASD-susceptibility genes impinge upon these processes (McFadden and Minshew 2013). Finally, it is worth noting that NRXN3 shows the lowest levels and the most restricted pattern of expression, and also the weakest association of gene polymorphisms with neurodevelopmental disorders (see Introduction section).

## Conclusion

Without discounting the importance of the contribution of NRXN mutations to synaptic dysfunction in neurodevelopmental conditions, the present study suggests that there may be potential roles for NRXNs in many fundamental developmental processes in the cerebral cortex. All three NRXNs are expressed at the earliest stages of human cortical development and show distinct expression patterns. Functional experiments should be designed to probe potential roles in cell migration and axon guidance, axon outgrowth, regulation of neuronal proliferation, and early development of subplate circuitry.

## Supplementary Material

Supplementary material are available at *Cerebral Cortex* online.

## Funding

The human fetal material was provided by the Joint UK MRC/Wellcome Trust (grant # 099175/Z/12/Z) Human Developmental Biology Resource (www.hdbr.org). The RNAseq study was funded by a grant from UK MRC (grant # MC/PC/13047).

## Supplementary Material

Supplementary Data

Supplementary Data

Supplementary Data

Supplementary Data
