# Supplementary figures and images for: Neurexins 1–3 Each Have a Distinct Pattern of Expression in the Early Developing Human Cerebral Cortex
Source: Cereb Cortex. 2016 Dec 24;27(1):216–32. doi: 10.1093/cercor/bhw394 (PMC5654756; doi:10.1093/cercor/bhw394)

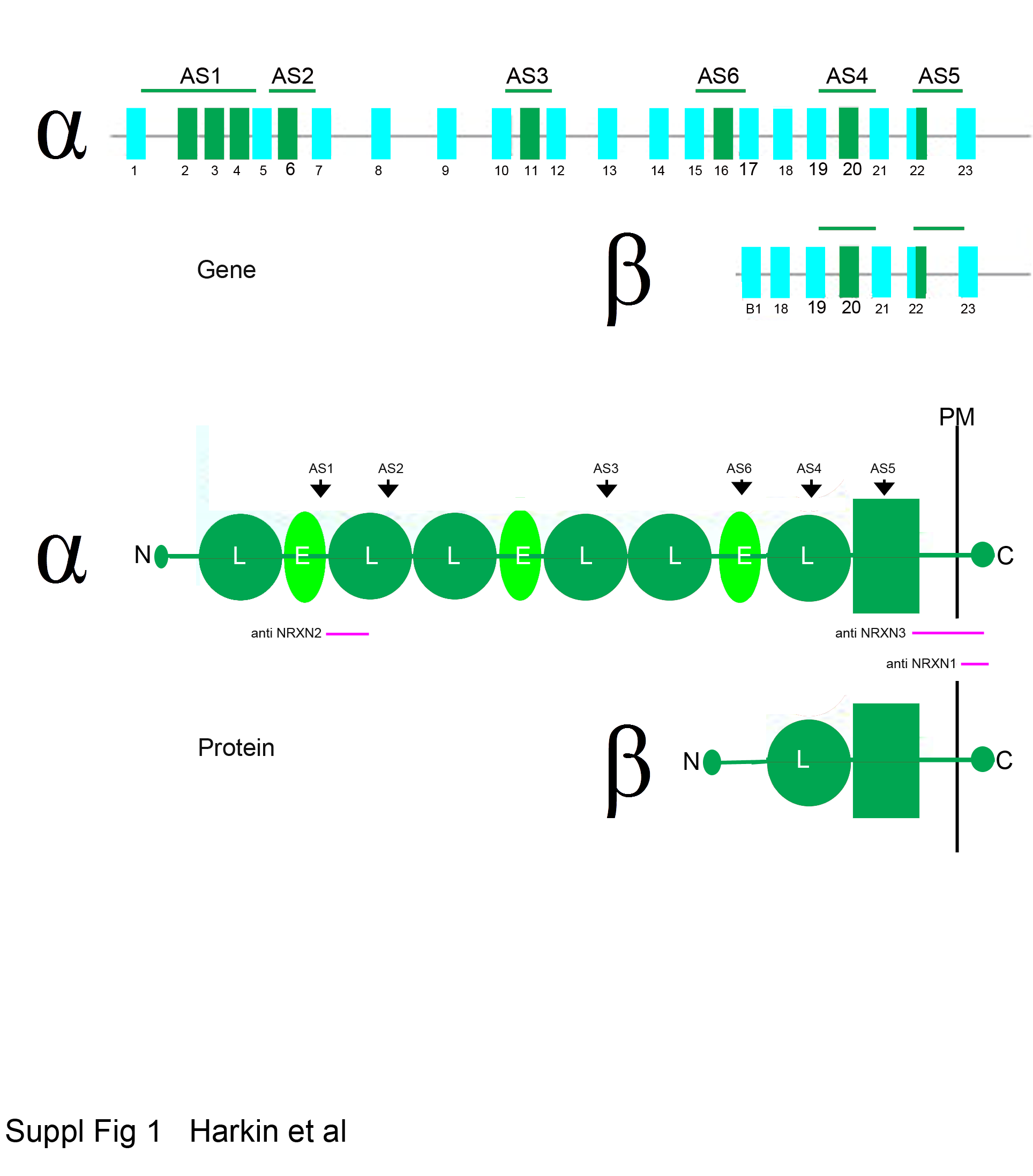

Supplement: Supplementary Data [file supplfig1.png]

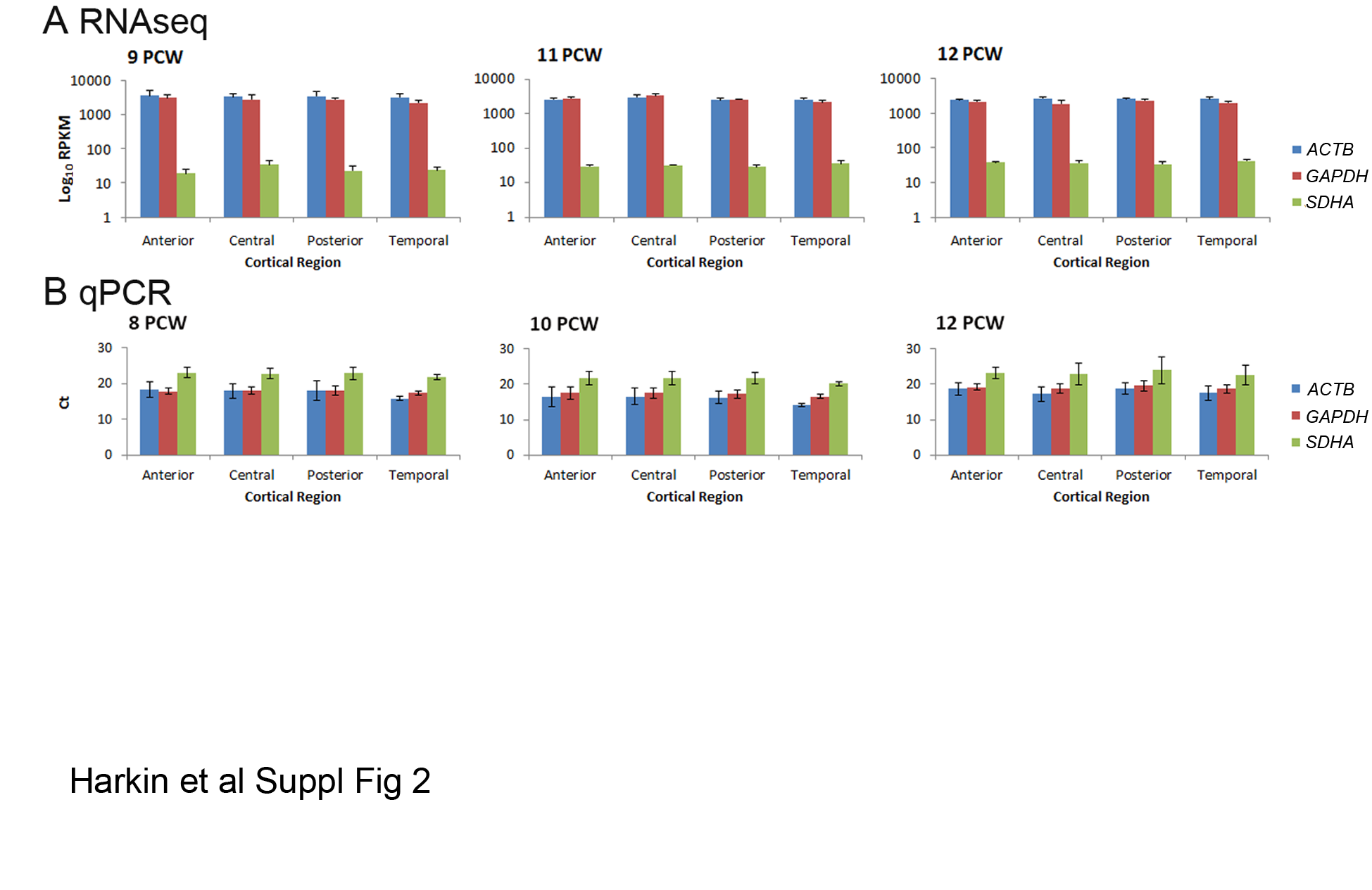

Supplement: Supplementary Data [file supplfig2.png]

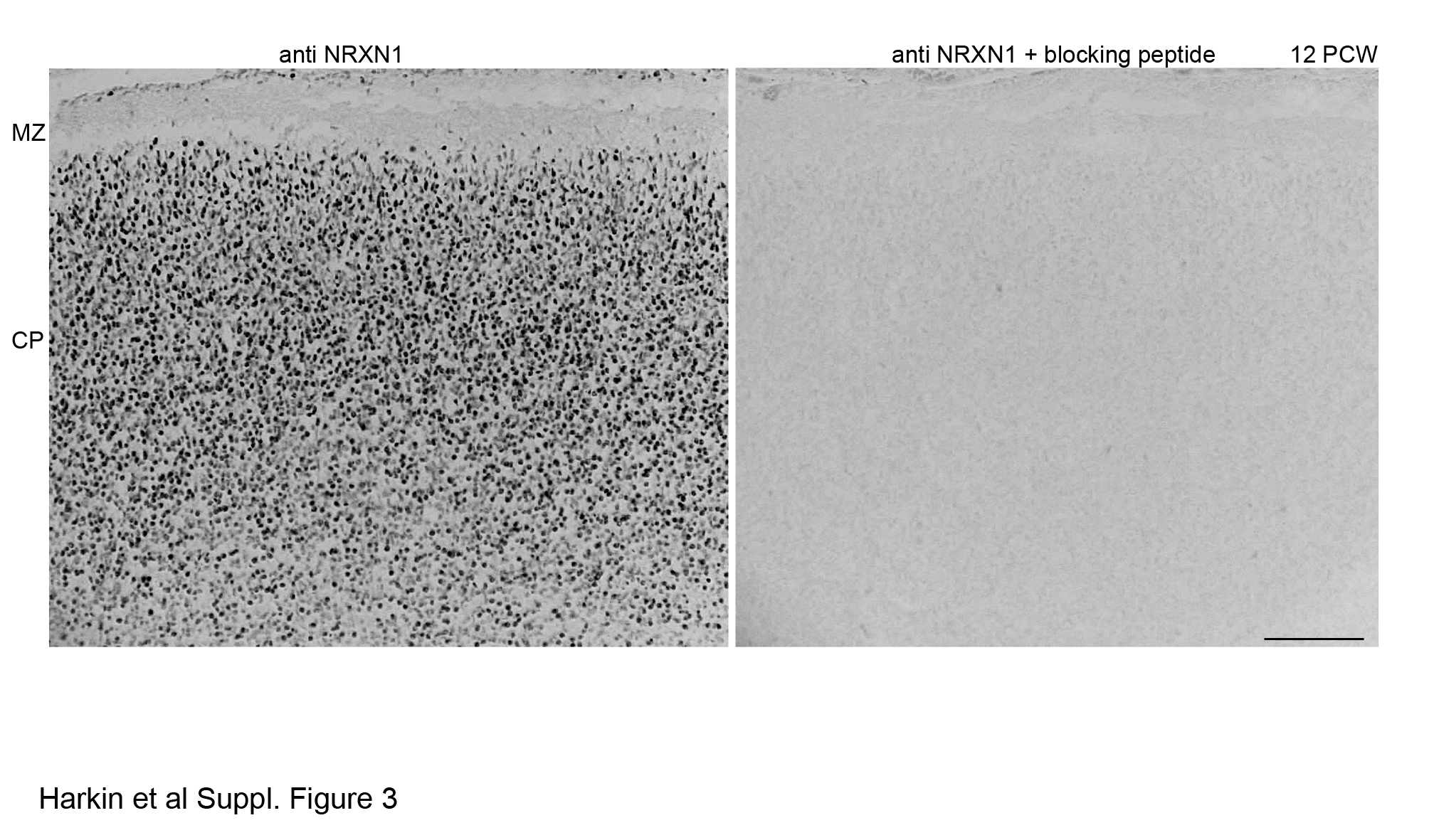

Supplement: Supplementary Data [file supplfig3r.png]
