# Supplementary material for: Neurexins 1–3 Each Have a Distinct Pattern of Expression in the Early Developing Human Cerebral Cortex
Source: Cereb Cortex. 2016 Dec 24;27(1):216–32. doi: 10.1093/cercor/bhw394 (PMC5654756; doi:10.1093/cercor/bhw394)
Supplement: Supplementary Data [file bhw394_supplementary_figure_legends.docx]

**Harkin et al.**

**Supplementary Figure 1**

A schematic showing the structure of the NRXN genes and proteins, indicating where the alternative splicing sites (AS) lie in relation to the exons and to the functional domains of the protein. The antigenic sites for each antibody used in this for immunohistochemistry are also marked. E, epidermal growth factor-like domain; L, laminin-neurexin-sex hormone binding globulin; PM, plasma membrane. Adapted from (Reissner et al, 2013; Schreiner et al, 2014)

**Supplementary Figure 2**

Expression of the reference genes *ACTB*, *GAPDH* and *SDHA* as measured by RNAseq and qPCR across all samples showing stable expression between cortical regions and across ages, demonstrating the appropriateness of using these genes for normalising expression of genes of interest. RPKM, Reads Per Kilobase of transcript per Million mapped reads; Ct, threshold cycle; PCW, post-conceptional weeks. Error bars indicate standard errors of the mean.

**Supplementary Figure 3**

Application of the blocking peptide led to the loss of all NRXN1 immunoreactivity in the section including nuclear staining. Sections from the cortical plate at 12 PCW.

Scale bar = 100 µm
